# Antilock braking system effectiveness in prevention of road traffic crashes in Iran

**DOI:** 10.1186/1471-2458-13-439

**Published:** 2013-05-04

**Authors:** Davoud Khorasani-Zavareh, Saeed Shoar, Soheil Saadat

**Affiliations:** 1 Social Determinants of Health Research Center, Urmia University of Medical Sciences, Urmia, Iran; 2 Department of Public Health Sciences, Division of Social Medicine, Karolinska Institutet, Stockholm, Sweden; 3 Students Scientific Research Center, Tehran University of Medical Sciences, Tehran, Iran; 4 Sina Trauma and Surgery Research Center, Tehran university of Medical Sciences, Tehran, Iran

## Abstract

**Background:**

Anti-lock Brake System (ABS) helps the equipped vehicles to stop under heavy braking, in a shorter distance and with a better control of direction. It was expected that this technology will reduce the rate of fatal road traffic crashes (RTC); however, the outcome is controversial in the real world. The aim of this study is to compare the claimed annual incidence rate and financial losses due to RTCs in ABS vs. non-ABS personal vehicles in Iran.

**Methods:**

A telephone survey among drivers of two similar models of personal vehicles was arranged. The studied vehicles were of the same brand and type; but only one of them was equipped with ABS. The number of RTCs, subsequent financial loss, and drivers’ knowledge and perception about ABS were sought. The sample consisted of drivers of 1232 ABS and 3123 non-ABS vehicles.

**Results:**

The annual incidence rate of RTC involving another vehicle was 145.1 (134.8-155.9) per 1000 vehicle-years and there was not a statistically significant difference between study groups.

The incidence of RTC with another vehicle due to brake failure was 50.3 (42.9-58.5) for 1000 non ABS vehicle-years and 30.0 (21.2-41.2) for 1000 ABS equipped vehicle-years. The difference was statistically significant after adjustment for the driver and vehicle's age and the daily driving time. The attributable risk of RTC for non-ABS vehicles was 20/1000 vehicles and the excess fraction was 39.8%. The mean financial loss due to reported RTCs was $987.9 ± $1547.3 US Dollars and there was not a statistically significant difference between study groups. While 61.1% of ABS vehicle drivers reported situations in which they believed the ABS had prevented a crash, 44.1% of them however, they did not know how to use ABS efficiently.

**Conclusions:**

Law enforcement to maintain safe distance and adhere to speed limit while driving, is needed to raise the effectiveness of ABS. This is as necessary as considering mandatory outfitting of ABS. Safety authorities should first consider the global experience and local evidence, before adopting any specific policy in this regard. The drivers need to learn the right way to use ABS for maximum effectiveness.

## Background

Being a major public health problem worldwide, Road Traffic Injuries (RTIs) are projected to increase in upcoming years [1]. RTI puts psychological and financial impacts on family and survivors as well [2]. Focusing on economic terms and gross national product (GNP), RTIs’ cost is estimated to be around 1% of GNP in low-income countries, while it is roughly 1.5% in middle-income and 2% in high-income countries [1].

RTI is a major health problem in Iran [3] with 114321 Road Traffic Crashes (RTCs) and 22976 fatal RTIs. The mortality rate of RTI is estimated as 31 per 100000 population in 2012, which ranks as the highest among middle-income countries for which reliable estimates can be made [2,4,5]. More than one percent of the Iranian population is affected as a result of RTIs annually, covering around one third of the Iranian hospitals beds. The Iranian parliament in 2004 lunched a new policy for reduction of RTIs in the country by 50 percent till end of 2015 [6]. Many activities including educational campaign, new law regulations with more focus on traffic calming, police enforcement on the new law as well as more restriction on graduated driver licensing programs in the country are lunched and updated in recent years [7,8]. The traffic safety culture and more importantly drivers’ attitude is still a major concern in Iran [9]. Moreover, transport system with more focus on vehicle safety promotion must take in to account to meet this new national policy [9]. The high burden of RTCs may be due to increasing of urbanization, a large land area, long distances between cities and its location at the crossroad of international trade routes [10].

Effective measures are needed to prevent RTCs and thus RTIs. These measures include Anti-lock Brake System (ABS) and Electronic Stability Control (ESC) system installation to prevent RTCs; seat belt, car seat and air bag to limit injury severity; and effective post-crash measures to minimize RTIs consequences [11]. Some road traffic safety regulatory authorities are going to consider the mandatory introduction of ESC systems, including ABS, as an effort to reduce RTCs [12,13]. ABS helps the equipped vehicles to stop under heavy braking, in a shorter distance, and with a much better control of direction than the conventional brake systems. In motor vehicles with conventional brake system, during a heavy braking or whenever the wheels begin to lock (such as seasonal iced or wet pavement), the force of the brake stopping the wheels exceeds the force making them rotate. Hence, such interference leads to skidding of the wheels which in turn, not only brings loss of directional control, but produces a very long stopping line in the pathway. However, the antilock brake system confronts the consistent force by reducing the pressure of liquid supplying the brake, so the brake force will increase just enough to a maximum level which is proper for stopping the car and in part not that much to lead to wheels lockup [14,15]. It was expected that this technology will reduce the rate of RTC and then the RTIs or obstacle avoidance, as was seen in crash tests [14,15]. However, the evidence was controversial in different studies [16-21].

Only a few vehicles are equipped with ABS in Iran and even in other countries [2,4]. The mandatory outfitting of new cars is under debate in Iran, considering the controversy mentioned above and the concerns about technical proficiency of local car manufacturers to provide effective and standard ABS for all new cars.

The aim of this study is to compare the claimed annual incidence rates and financial losses of RTC in ABS equipped vs. conventional brake systems personal cars in Iran.

## Methods

This study was conducted in Iran. The total number of registered vehicles was about 14 million and the number of vehicles per 1000 inhabitants was equal to 13.

The study design was historical cohort. The study population was the drivers of two similar vehicles of the same manufacturer and similar engine, design and price. The reason for restricting the study to cars of only one manufacturer was to limit the sources of variation such as socioeconomic status of driver and the technical differences. The main difference between the studied vehicles was the ABS installation. The unexposed groups’ vehicles were equipped with ABS while the exposed group had the traditional brake system.

Study period was March 2007 to March 2008 (one complete Persian calendar year). We contacted on the cell phones of the study subjects at the end of the Persian year and asked them to report their age (years), sex, the frequency of intercity trips, the number of people who drove the vehicle and the vehicle’s age (Additional file 1). They were asked to report the mean daily driving time (DDT) in hours and their answers were verified according to the frequency of gas fillings.

The number of RTCs during the past Persian calendar period and the information on the reported collisions including the cause, financial toll, injuries and the role of brake failure (according to their perception) were sought during a structured telephone interview. Accordingly, crashes due to brake failure were defined as “crashes that could potentially be avoided if the brakes stopped the cars faster or if cars did not skid while braking”. The drivers of unexposed group were asked to report if there have been situations that ABS had prevented a traffic crash. They were also asked to report how they usually used the (ABS) brake, to explore their knowledge of the right way to use ABS.

The power of study to detect a 10%, 20% and 30% difference in the incidence rate of RTCs in the ABS compared to non-ABS vehicles was 0.20, 0.65, and 0.96 respectively.

### Case selection

The sample consisted of drivers of 1400 ABS equipped, and 3500 conventional brake system personal cars. They were selected using simple random sampling method among the registry of the Central Insurance Organization. The response rate was 88.0% (1232 out of 1400) and 89.2% (3123 out of 3500) for ABS and non-ABS group, respectively. The inclusion criterion was as follows: being a driver of the selected car during the study period. Informants those who were not the main driver of the studied vehicle and those who declined to participate in the study were excluded.

### Data treatment

The mean and standard deviation (SD) were calculated for continuous data; and student’s *t*-test was used for comparing continuous variables. The relative frequency of categorical variables was calculated as percentage; and Chi-square test was used for between groups comparison. The incidence rates are reported as point estimate and 95% confidence interval; the Poisson distribution assumption was used to calculate the confidence interval. P-value < 0.05 was considered as statistically significant. Poisson regression analysis was used to compare the number of RTCs due to brake failure (outcome variable) among ABS vs. conventional vehicles, controlling for the effect of driver’s age, DDT, car age, number of drivers, and the frequency of intercity trips (dependent variables). The variables that did not attain statistical significance in the model were later excluded. The STATA version 8.00 SE was used for data analysis.

The study was ethically approved by Sina Trauma and Surgery Research Centre affiliated to Tehran University of Medical Sciences.

## Results

**Figure 1:**
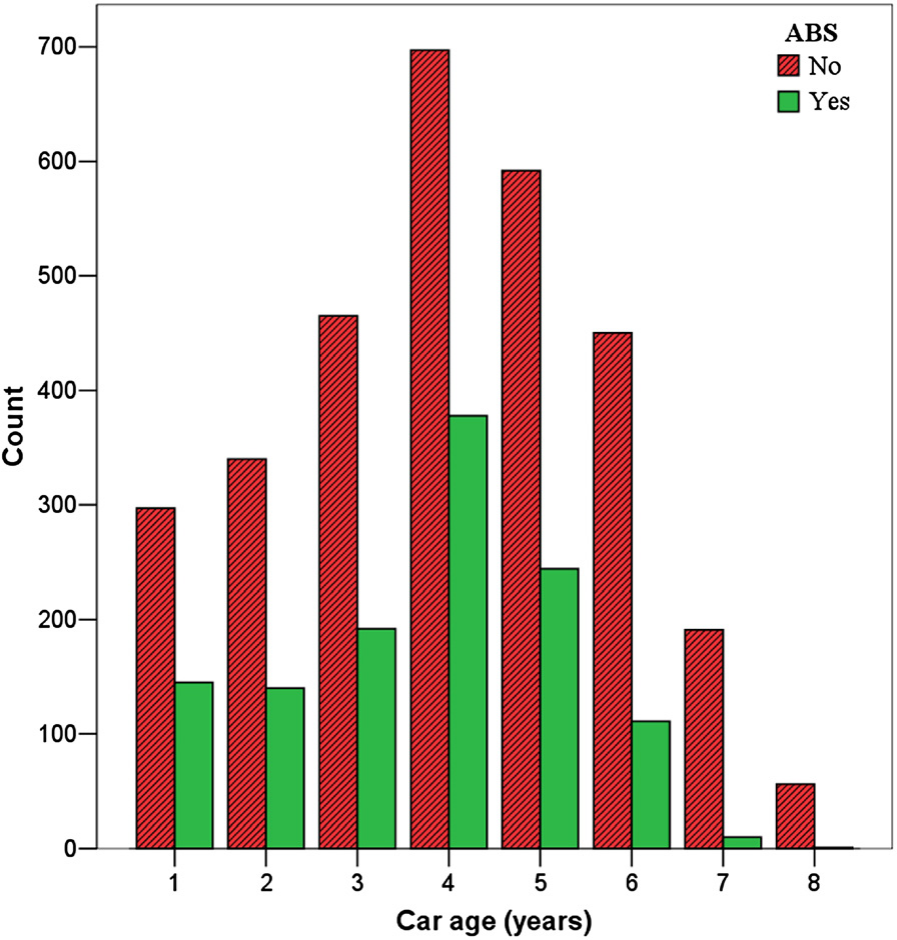
The mean age of ABS versus non-ABS vehicles in the study.

In total, 795 (18.2%) of responders were female, with a male-to-female ratio of 4.9/1. The mean age of drivers was 40.3 ± 10.3 (median: 39.0) years and the DDT was 2.1 ± 1.9 (median: 2.0) hours (See Table 1). The mean age of cars equipped with ABS was 3.7 ± 1.5 and cars without ABS was 4.1 ± 1.8 years (P < 0.001) (Figure 1). Among all reported crashes, 75% had occurred in urban areas.

**Table 1:** Demographics of study subjects

**Demographics**	**Mean (±SD)**	**Median**
Age of drivers (years)	40(±10.0)	39.0
Daily driving time (hours)	2.2(±1.9)	2.0
Number of intercity trips per week	3.9(±2.3)	3.5
Age of car (years)	4.0(±1.7)	4.0

SD: Standard Deviation.

In 2323 (53.1%) cases, the cars were exclusively driven by the responding driver; however, in 1849 (42.3%) cases, the cars were reported to be driven by another driver as well. The remainder 4.6% of the studied vehicles had more than three drivers. Among ABS vehicle drivers, 61.1% reported situations in which they believed ABS had prevented a crash. On the other hand, 44.1% of ABS vehicle drivers did not know the right way to use ABS.

The incidence rate of all, injurious and fatal traffic crashes were 145.1 (134.8-155.9), 9.6 (7.0- 13.0), and 0.5 (0.1-1.7) per 1000 vehicles (of studied type), respectively. The relative frequency of reported injuries due to RTC were as follows: bone fracture (42.3%), superficial (23.1%), contusion (15.4%), head injury (11.5%), amputation (3.8%), and internal bleeding (3.8%).

The mean financial loss due to reported RTCs was $987.9 ± $1547.3 United States (US) dollars (Median: $538.6; Range: $21.5-$26930.9). The average exchange rate during the study period was US$ 1.00 = 9283 Iranian Rials [22]. There was not a statistically significant difference among study groups (Table 2).

**Table 2:** Comparison of RTCs and their location and financial toll in ABS versus non-ABS personal cars

**Variables**	**Category**	**ABS**	**Percentage**	**Non-ABS**	**Percentage**
Total RTC	0	1059	86.0%	2708	86.7%
	1	158	12.8%	391	12.5%
	2	12	1.0%	22	0.7%
	3	3	0.2%	2	0.1%
Injurious RTC	0	1223	99.3%	3099	99.2%
	1	5	0.4%	21	0.7%
	2	3	0.2%	2	<0.1%
	3	1	0.1%	1	<0.1%
Fatal RTC	0	1232	100.0%	3121	99.9%
	1	0	0.0%	2	0.1%
Locality of RTC	Urban	151	79.1%	324	73.5%
	intercity	40	20.9%	117	26.5%
RTCs due to the brake failure	0	1195	97.0%	2971	95.1%
	1	37	3.0%	147	4.7%
	2	0	0.0%	5	0.2%
Financial losses due to RTC (Mean ± SD; median)	1179.9 ± 2377.4; 646.3			916.5 ± 1084.2; 538.6	

### All crashes

The overall annual incidence rate of RTC involving another vehicle was 145.1 (134.8-155.9) per 1000 vehicle-years. There was not a statistically significant difference in the reported incidence rate of RTC in ABS vs. conventional brake system vehicles (P = 0.39). Moreover, the difference failed to attain statistical significance in Poisson regression analysis after adjustment for the effects of age of driver, age of vehicle, number of drivers, DDT and the frequency of intercity trips.

The incidence rate of death and injury per 1000 RTC were 12.6 (1.5-45.4) and 69.2 (34.5-123.8) for conventional brake systems vehicles; and 0 (0–97.0) and 26.3 (0.6-146.6) for ABS equipped vehicles, respectively. The difference was not statistically significant.

The incidence rate of hitting a pedestrian by a car was 5.3 (3.4-7.9) per 1000 vehicles and there was not a statistically significant difference between ABS vs. conventional brake system vehicles.

### Crashes due to brake failure

The incidence rate of RTC due to brake failure with another vehicle was 50.3 (42.9-58.5) for 1000 conventional brake system vehicle-years vs. 30.3 (21.2-41.2) for 1000 ABS equipped vehicle-years (P < 0.01). The attributable risk of RTC for conventional brake systems vehicles compared to ABS vehicles was 20.0 (7.7-32.3) per 1000 vehicles, and the excess fraction was 39.8% (14.4%-57.7%). Table 3 represents the association of ABS with the number of RTC due to brake failure, controlling the effect of other variables, using Poisson regression model. Number of drivers and the frequency of intercity trips are excluded from the model because they did not attain statistical significance in the model.

**Table 3:** Association of ABS with RTCs due to brake failure, controlling the effect of potential confounders, reduced model

**Variable**	**Coefficient**	**SE**	**95% Confidence interval**	**P value**
ABS	−0.55	0.19	(−0.92 - −0.18)	0.004
Driver’s age (years)	−0.02	0.01	(−0.04 - −0.01)	0.003
DDT (hours)	0.10	0.03	(0.05 - 0.16)	<0.001
Car age (years)	0.09	0.04	(0.01 - 0.17)	0.045
Model constant	−1.96	0.36	--	<0.001

SE: Standard Error.

Goodness of fit statistics: Deviance = 1133.9; degree of freedom (df) =4080; Value/df = 0.28.

Likelihood ratio Chi Square = 29.1; df = 3; p-value < 0.001.

## Discussion

This study is the first one in the region, to our knowledge, that gauges the effectiveness of the ABS in practice and in Iran climate. The annual incidence rate of RTC in this study was similar to population based estimates in Iran [23]; therefore, the sample included in this study could be considered a representative sample of Iranian drivers. Drivers of ABS equipped vehicles reported less RTCs due to brake failure compared to similar vehicles that were not equipped with ABS. However, there was not a difference in the annual incidence rates of total, injurious and fatal RTCs between the two groups.

A 39.8% excess fraction for RTCs due to brake failure in non-ABS vehicles indicates usefulness of ABS to prevent RTCs. However, lack of support for the difference by objective outcome measures such as actual RTCs raise a question on effectiveness of ABS in Iran. On the other hand, poor knowledge of drivers of ABS equipped vehicles about the right way to use ABS may invalidate their judgment about the effectiveness of ABS in preventing RTCs due to brake failure. This indicates the need for training of drivers of ABS equipped vehicles.

The power of this study indicates that it is highly unlikely that ABS had decreased the rate of RTCs to 30%. This might be a result of low effectiveness of ABS in current cars as well as incomplete knowledge of drivers about the right way to use ABS. Another explanation would be careless driving by ABS equipped vehicles, due to overestimation of ABS effectiveness. Drivers may have not maintained the safe distance from the car in front of them, as a result of over confidence on ABS. Failure to keep safe distance is a common offence in Iran and traffic police appears tolerant about that. Although ABS stops the vehicle in shorter distance; however, failure to keep the safe distance could easily vanish the advantage of ABS technology. The enforcement of traffic law, especially in case of keeping safe distance and speed limit, seems as necessary as mandatory outfitting of ABS.

It has been expected that ABS technology will reduce the rate of RTCs and RTIs, as was seen in track tests [14,15]. However, the findings have been inconsistent in the real field [16-21]. The Highway Loss Data Institute (HLDI) in the U.S. has shown that no change in claim frequency had been observed after adding the ABS technology [24]. The incidence rate of single-vehicle crashes in ABS equipped cars was reported high while it had been diminished in multiple-vehicle setting [25].

There are reports indicating decreased RTIs and increased fatal RTCs involving vehicles with ABS [17,18,26,27]. Moreover, a significant increase in overturning crash, single vehicle crash and collisions with fixed objects has been attributed to the ABS [20,26,28]. While Evans and Gerrish proposed the risk compensation as an explanation [28], Kahane claimed improper operation of ABS equipped vehicles as the reason for failure of the ABS to prevent all forms of RTCs [19], which was supported by Harless study in 2002 [26]. Improper usage of ABS may be another reason that prevents ABS to appear as effective as expected. Forty four percent of drivers of ABS equipped vehicles in our study either released the brake pedal when they felt “a sense of crashing under their feet”, or pumped the pedal in a same way as they did in conventional brakes. This is in line with a study conducted in North Carolina and Wisconsin which showed that close to half of drivers did not have the knowledge of ABS use [25]. This is more interesting when considering the positive effect of knowledge improvement, which has been seen after training the drivers to use ABS by the transfer of verbal knowledge [1]. However, at this time, there is no training for drivers whose vehicles are equipped with ABS in Iran. It seems necessary to provide comprehensible information and training about how to use ABS for optimal performance and how ABS could improve the safety while braking.

Car manufacturers in Iran have been recently sought to install ABS for all their cars. However, there are concerns about proper operation of ABS when outfitted into cars for which they were not originally designed for. While mandatory outfitting of all cars with ABS can improve safety, it is noteworthy to consider the cost effectiveness of this policy. The benefit to cost ratio of ABS installation of vehicle has been reported as 0.7 in Norway [29]. This ratio has been reported as 1.3 for child restraints, 3.3 for mandatory daytime running lights for cars, 16.7 for vehicle crashworthiness in cars by using collapsible steering columns, and 31.7 for safety seat belts of drivers. Safety authorities in Iran should first invest to prepare a list of safety measures priorities, considering the global experience and local evidence, before adopting any specific policy regarding mandatory fitting of ABS in vehicles. This study could be considered as local evidence; however, it needs to be repeated with additional sample size in different settings, i.e. diverse climates and types of vehicles.

### Limitations and strengths of study

This study asked the drivers to report their car crashes due to brake failure. Drivers of non-ABS vehicles might have believed that “if their car had been equipped with ABS, then it would not have crashed, and therefore probably overestimated “the crashes due to brake failure”. On the other hand, drivers of ABS equipped vehicles might have expected a lot from the ABS; therefore, overestimating “the crashes due to brake failure”. Therefore, the overestimation in one group may have been compensated in the other group; however we were not able to measure it.

## Conclusions

While ABS is believed to prevent RTCs due to brake failure (up to 40%), its cost-effectiveness needs to be evaluated in different settings with different type of vehicles. Road safety authorities in Iran should first consider the global experience and local evidence in safety equipment priorities, before adopting any specific policy vis-à-vis mandatory ABS in vehicles. ABS will be of limited value if drivers fail to keep safe distance and adhere to speed limits; therefore, traffic authorities should give higher priority to law enforcement, when they consider mandatory outfitting of ABS. In addition, the drivers need to learn the right way to use ABS for maximum effectiveness. This could be facilitated by the organizations responsible for the road traffic safety in the country.

## Competing interests

There is no conflict of interest in any forms which could affect this study.

## Authors’ contributions

SS designed the study, managed data collection, participated in data analysis, interpretation and drafting of the manuscript. DKZ participated in data analysis, interpretation and drafting the manuscript. SSh participated in drafting the manuscript and literature review. All authors read and approved the final manuscript.

## Pre-publication history

The pre-publication history for this paper can be accessed here:

http://www.biomedcentral.com/1471-2458/13/439/prepub

## Supplementary Material

Additional file 1 The data collection form. Click here for file
