# Supplementary material for: Antilock braking system effectiveness in prevention of road traffic crashes in Iran
Source: BMC Public Health. 2013 May 4;13:439. doi: 10.1186/1471-2458-13-439 (PMC3649935; doi:10.1186/1471-2458-13-439)
Supplement: Additional file 1 — The data collection form. [file 1471-2458-13-439-S1.doc]

Sina Trauma and Surgery Research Centre

Serial Number:

The association of ABS and Road Traffic Collision Study, Data Collection form 1

City:

ABS: Yes No

Name of owner:

1. Who is the main driver of your vehicle?

Continue the interview if the responder is the main driver!

Myself Spouse Children Others

1. How old are you (in years)?
2. How old is your car (in years)?
3. Your gender please: Male Female
4. How many times did you experience a traffic collision with another vehicle during the past calendar period?
   1. Please specify the situation in the form 2.
5. How many times did you hit to a pedestrian during the past calendar period?
   1. Please specify the situation in the form 2.
6. Are there any other persons who drive your car occasionally? How many persons?
7. How many hours do you drive a day in average?
8. How often do you re-fill the tank?
9. How often do you drive for an “intercity trip” by this car? every _______ days
10. How many times did you go to a travel by this car during the past calendar period?
11. Did you experience any traffic collision that was due to brake failure during the past calendar period?
    1. Please specify the situation in the form 2.

The following questions should be asked from drivers of ABS equipped vehicles only!

1. Did you experience a situation in which ABS prevented a traffic collision?
   1. If yes, proceed to form 2.
2. How do you brake if you are driving in high speed on slippery road and you have to stop promptly?
